# Effect of a freeze–thaw cycle on amniotic fluid interleukin-6 concentrations in pregnancies with preterm prelabor rupture of membranes: Implications for the diagnosis of intra-amniotic inflammation

**DOI:** 10.1371/journal.pone.0347105

**Published:** 2026-06-11

**Authors:** Richard Spacek, Stepanka Bubenikova, Ivana Musilova, Nikola Kuzniciusova, Karolina Janochova, Michaela Kostelkova, Ladislava Pavlikova, Magdalena Holeckova, Marek Lubusky, Ondrej Simetka, Bo Jacobsson, Marian Kacerovsky

**Affiliations:** 1 Department of Obstetrics and Gynecology, University Hospital Ostrava, Faculty of Medicine in Ostrava, Ostrava, Czech Republic; 2 Department of Midwifery, Palacky University, Faculty of Health Sciences, Olomouc, Czech Republic; 3 Biomedical Research Center, University Hospital Hradec Kralove, Hradec Kralove, Czech Republic; 4 PRENET, Center for prenatal diagnostic, Pardubice, Czech Republic; 5 Institute of Laboratory Medicine, University Hospital Ostrava, Faculty of Medicine in Ostrava, Ostrava, Czech Republic; 6 Institute of Clinical Biochemistry and Diagnostics, University Hospital Hradec Kralove, Charles University, Faculty of Medicine in Hradec Kralove, Hradec Kralove, Czech Republic; 7 Department of Obstetrics and Gynecology, University Hospital Olomouc, Palacky University, Faculty of Medicine and Dentistry in Olomouc, Olomouc, Czech Republic; 8 Department of Obstetrics and Gynecology, Institute of Clinical Science, Sahlgrenska Academy, University of Gothenburg, Gothenburg, Sweden; 9 Department of Obstetrics and Gynecology, Sahlgrenska University Hospital, Gothenburg, Västra Götaland region, Sweden; 10 Department of Genetics and Bioinformatics, Domain of Health Data and Digitalization, Institute of Public Health, Oslo, Norway; University of Health Sciences (Istanbul, Türkiye), TÜRKIYE

## Abstract

**Objective:**

Preterm prelabor rupture of membranes (PPROM) is frequently complicated by intra-amniotic inflammation. Interleukin-6 (IL-6) measured in amniotic fluid is considered the gold-standard biomarker for the diagnosis of this condition; however, diagnostic thresholds have been derived primarily from biobanked samples. It remains unclear whether IL-6 concentrations measured in fresh amniotic fluid are directly comparable with those obtained from previously processed samples. The primary aim of this study was to compare IL-6 concentrations in fresh amniotic fluid samples with those in biobanked samples that had undergone a single freeze–thaw cycle.

**Method:**

This retrospective study included consecutive singleton pregnancies with PPROM between 24 + 0 and 36 + 6 weeks of gestation. Amniotic fluid samples were collected on admission, prior to the administration of antibiotics and corticosteroids. IL-6 concentrations were measured in both fresh and biobanked (processed) samples using an automated electrochemiluminescence immunoassay.

**Results:**

The study population comprised 152 women. IL-6 concentrations in fresh and processed samples were strongly correlated (rho = 0.97; p < 0.0001). Bland–Altman analysis demonstrated a systematic bias toward higher concentrations in processed samples, with a mean ratio of 1.2, indicating that processed samples yielded on average approximately 20% higher values than fresh samples. When applying the validated diagnostic threshold of 3,000 pg/mL (established in processed samples) to fresh samples, three false-negative cases were observed. In these cases, intra-amniotic inflammation was present, but IL-6 concentrations in fresh samples were below the threshold despite corresponding processed values ≥ 3,000 pg/mL. Using intra-amniotic inflammation defined by processed samples as the reference, the diagnostic performance of the 3,000 pg/mL threshold applied to fresh samples was as follows: sensitivity 92%, specificity 100%, positive predictive value 100%, and negative predictive value 97%.

**Conclusions:**

IL-6 concentrations in fresh and processed amniotic fluid samples are highly correlated, but processed samples consistently yield higher values.

## Introduction

Preterm prelabor rupture of membranes (PPROM), defined as rupture of the fetal membranes before 37 weeks of gestation and prior to the onset of labor [1,2]. PPROM represents approximately one-third of all preterm birth and contributes substantially to neonatal morbidity and mortality worldwide [1,2]. A considerable proportion of PPROM cases are complicated by intra-amniotic inflammation, a condition strongly associated with adverse pregnancy outcomes, including preterm delivery, neonatal sepsis, and long-term neurodevelopmental impairment [3–5].

Recent studies suggest that individualized management of PPROM based on the presence or absence of intra-amniotic inflammation is associated with improved maternal and neonatal outcomes compared with uniform expectant treatment strategy [6,7]. Reliable identification of intra-amniotic inflammation is therefore essential for guiding personalized clinical decision-making.

Interleukin (IL)-6 has emerged as the gold-standard biomarker for the diagnosis of intra-amniotic inflammation [8–10]. Several diagnostic thresholds have been established, depending on the method of measurement: 2.6 ng/mL for enzyme-linked immunosorbent assay (ELISA) [11], 0.745 ng/mL for the Millenia point-of-care test [12,13], and 3.0 ng/mL for automated electrochemiluminescence immunoassay [14]. Importantly, these cut-offs were derived from analyses of biobanked amniotic fluid samples that had undergone at least one freeze–thaw cycle [8,12–14].

In clinical practice, however, IL-6 concentrations are measured in fresh amniotic fluid obtained immediately after sampling [9,15,16]. It still remains unclear whether IL-6 concentrations in fresh and previously processed samples are comparable, and whether diagnostic thresholds derived from frozen/thawed specimens can be directly applied to fresh clinical samples. To address this knowledge gap, the main aim of this study was to compare IL-6 concentrations in fresh amniotic fluid samples with those in biobanked samples that had undergone a freeze–thaw cycle in women with PPROM, and to assess the implications for the diagnosis of intra-amniotic inflammation. Both measurements were performed in a high-volume biochemistry laboratory using an automated electrochemiluminescence immunoassay to ensure clinical relevance.

## Materials and methods

This retrospective cohort study was conducted at the Department of Obstetrics and Gynecology, University Hospital Hradec Kralove, Czech Republic. Participants were pregnant women admitted between January 1, 2021, and June 30, 2023. Inclusion criteria were: a) singleton pregnancy; b) maternal age ≥ 18 years; c) PPROM at 24 + 0–36 + 6 gestational weeks; and d) transabdominal amniocentesis performed at admission to assess intra-amniotic inflammation. Exclusion criteria were: a) medical or obstetric complications (e.g., fetal growth restriction, pre-gestational or gestational diabetes, chronic or gestational hypertension and preeclampsia; b) chromosomal or structural fetal abnormality; c) signs of fetal hypoxia; and d) significant vaginal bleeding.

Gestational age was determined based on the first trimester fetal biometry. PPROM diagnosis was established by sterile speculum examination to identify pooling of amniotic fluid in the posterior vaginal fornix. In cases of diagnostic uncertainty, amniotic fluid leakage was confirmed by testing vaginal fluid for insulin-like growth factor-binding proteins (Actim PROM test; Medix Biochemica, Kauniainen, Finland) [17,18].

Women with PPROM received intravenous antibiotics: benzylpenicillin (or clindamycin in case of penicillin allergy) if intra-amniotic inflammation was absent, and clarithromycin if intra-amniotic inflammation was present. Antibiotic therapy was continued for seven days unless delivery occurred earlier.

Women with PPROM and intra-amniotic infection at gestational age > 28 + 0 weeks underwent induction of labor or elective cesarean section within 72 hours of admission. Remaining women with PPROM were managed expectantly. Women with PPROM at < 35 + 0 weeks’ gestation were given intramuscular betamethasone to accelerate fetal lung maturation and reduce neonatal morbidity and mortality. During active labor, women with PPROM who had positive vaginal-rectal swabs for *Streptococcus agalactiae*, or an unknown colonization status, received intravenous benzylpenicillin (or clindamycin, in case of penicillin allergy).

All women were Caucasian and provided written informed consent prior to sampling. Ethical approval for the collection of leftovers of amniotic fluid for research, was granted by the Institutional Review Board of the University Hospital Hradec Kralove (February 2019, No. 201902 S16P). The study itself was approved by the Institutional Review Board of University Hospital Hradec Kralove (June 2025, No. 202506 P05). Prior to analysis, which began September 1, 2025, all data were fully anonymized. Information that could identify participants during or after data collection was accessible only to the study authors. All sampling and analyses were performed in accordance with the relevant guidelines and regulations. Aliquots of these samples were used in our previous studies [19–21]

### Amniotic fluid sampling and analysis

Amniotic fluid was obtained via transabdominal amniocentesis. Each sample was divided into 0.5 mL aliquots, which were immediately sent for: i) assessment of IL-6 concentrations using an automated electrochemiluminescence immunoassay method (fresh amniotic fluid); ii) aerobic and anaerobic cultivation; iii) species-specific polymerase chain reaction (PCR) to detect *Ureaplasma* spp., *Mycoplasma hominis*, and *Chlamydia trachomatis* DNA; and iv) species-nonspecific PCR targeting the 16S rRNA gene, followed by Sanger sequencing. The remaining amniotic fluid was processed (processed amniotic fluid) and stored at −80°C until analyses.

### Amniotic fluid IL-6 concentrations in fresh and processed samples

**Fresh amniotic fluid** samples were delivered immediately after collection to a high-volume biochemistry laboratory, where they were centrifuged for 5 minutes at 2000 *g* at 4°C to remove cells and debris, and then analyzed.

**Processed amniotic fluid** samples were placed into refrigerator (4°C) immediately after collection and centrifuged within one hour for 15 minutes at 2000 *g* at 4°C to remove cells and debris. The samples were then divided into the 0.5 mL aliquots and stored at −80°C until analyses. For this study, only aliquots subjected to a single freeze-thaw cycle were used.

IL-6 concentrations in both fresh and processed samples were determined using a Cobas e602 immunoanalyzer, part of the Cobas 8000 platform (Roche Diagnostics, Basel, Switzerland). The measuring range was 1.5–5,000 pg/mL, extendable to 50,000 pg/mL, with 10-fold dilution. Samples with IL-6 concentrations > 50,000 pg/mL were assigned a value of 50,001 pg/mL [14]. The coefficient of variation for inter-assay and intra-assay precisions were < 10% [14].

### Detection of Ureaplasma spp., M. hominis, C. trachomatis and other bacteria in amniotic fluid

The commercial AmpliSens^®^ *C. trachomatis*/*Ureaplasma*/*M. hominis*-FRT kit (Federal State Institution of Science, Central Research Institute of Epidemiology, Moscow, Russia) was used to detect DNA of *Ureaplasma* spp., *M. hominis* and *C. trachomatis*. Details of the detection of other bacteria in amniotic fluid by sequencing of the 16S rRNA gene and by aerobic/anaerobic cultures have been described previously [17].

### Clinical definitions

**Intra-amniotic inflammation**: interleukin-6 concentration in fresh amniotic fluid **≥** 3000 pg/mL. **Microbial invasion of the amniotic cavity:** presence of microorganisms and/or their nucleic acids in amniotic fluid. **Intra-amniotic infection**: concomitant presence of both intra-amniotic inflammation and microbial invasion of the amniotic cavity. **Sterile intra-amniotic inflammation**: presence of intra-amniotic inflammation without microbial invasion of the amniotic cavity. **Colonization of the amniotic cavity:** presence of microbial invasion of the amniotic cavity without intra-amniotic inflammation. **Negative amniotic fluid:** absence of both intra-amniotic inflammation and microbial invasion of the amniotic cavity

### Statistics

The nonparametric Kruskal-Wallis *H* test was applied to compare continuous variables (presented as median values; interquartile range [IQR]) among demographic and clinical characteristics. Categorical variables, presented as numbers (%), were compared using Chi-square test. Normality of data was assessed with the Anderson–Darling test. Amniotic fluid IL-6 concentrations in fresh and processed samples were compared with the paired Wilcoxon test. Spearman’s correlation coefficient was used to assess the correlation between amniotic fluid IL-6 concentrations in fresh and processed samples. A ratio-based Bland-Altman plot was constructed to assess agreement between amniotic fluid IL-6 concentrations in fresh and processed samples. Log transformation was not applied, as IL-6 concentrations are used directly in clinical practice to rule in or rule out intra-amniotic inflammation based on absolute threshold. Sensitivity analyses excluding samples with IL-6 concentrations above the upper measuring limit were performed to assess the robustness of correlation, paired comparison, and Bland-Altman analyses. All *p* values were two-tailed, and differences were regarded as statistically significant at *p* < 0.05. All statistical analyses were performed using GraphPad Prism, version 10.6.1 for Mac OS X (GraphPad Software, San Diego, CA, USA).

## Results

A total of 192 women with late PPROM were recruited. Forty women were excluded due to gestation diabetes mellitus (n = 25), fetal growth restriction (n = 6), preeclampsia (n = 5), gestational hypertension (n = 2), and chronic hypertension (n = 2). The final study population therefore comprised 152 women.

Among the included women, 24% (36/152) had intra-amniotic inflammation and 26% (40/152) microbial invasion of the amniotic cavity. When women were stratified into groups according to the presence or absence of these conditions, 18% (27/152) had intra-amniotic infection, 6% (9/152) had sterile intra-amniotic inflammation, 9% (13/152) had colonization of the amniotic cavity, and 67% (103/152) had negative amniotic fluid for both inflammation and microorganisms. The demographic and clinical characteristics of the women and their neonates according to these groups are summarized and 26% (40/152) in Table 1.

**Table 1:** Demographic and clinical characteristics of women with preterm prelabor rupture of membranes (PPROM) based on the presence and absence of intra-amniotic infection, sterile intra-amniotic inflammation, colonization of the amniotic cavity, and negative amniotic fluid.

	Intra-amniotic infection (n = 27)	Sterile intra-amniotic inflammation (n = 9)	Colonization of the amniotic cavity (n = 13)	Negative amniotic fluid (n = 103)	*p-*value
Maternal age [years, median (IQR)]	32 (28-36)	29 (27-31)	30 (26-33)	30 (27-35)	0.30
Primiparous [number (%)]	9 (33%)	8 (89%)	5 (39%)	53 (51%)	**0.03**
Smoking [number (%)]	6 (22%)	2 (22%)	4 (31%)	7 (7%)	**0.02**
Pre-pregnancy body mass index [kg/m^2^, median (IQR)]	24.6 (20.2-30.1)	21.7 (21.2-29.2)	23.8 (20.4-26.6)	25.2 (21.4-30.3)	0.51
Gestational age at sampling [weeks + days, median (IQR)]	29 + 4 (29 + 0-30 + 5)	30 + 0 (27 + 5-34 + 2)	33 + 0 (30 + 2-34 + 3)	34 + 1 (31 + 0-35 + 1)	**<0.0001**
Gestational age at delivery [weeks + days, median (IQR)]	31 + 0 (29 + 4-31 + 4)	30 + 6 (30 + 0-34 + 3)	33 + 2 (31 + 4-35 + 0)	34 + 3 (32 + 1-35 + 4)	**<0.0001**
Latency from PPROM to amniocentesis [hours, median (IQR)]	5 (3-10)	6 (2-9)	4 (3-4)	4 (2-6)	0.12
Latency from PPROM to delivery [hours, median (IQR)]	196 (74-300)	52 (29-150)	62 (32-186)	51 (22-258)	0.22
CRP levels at admission [mg/L, median (IQR)]	10.7 (4.2-21.4)	4.9 (2.8-15.6)	3.4 (2.2-8.6)	4.6 (2.5-7.9)	**0.003**
WBC count at admission [x10^9^ L, median (IQR)]	12.4 (10.6-14.3)	14.4 (10.9-17.1)	10.4 (9.9-12.8)	10.7 (8.8-12.8)	**0.02**
Administration of corticosteroids [number (%)]	25 (93%)	7 (78%)	10 (77%)	55 (53%)	**0.001**
Administration of antibiotics [number (%)]	26 (96%)	9 (100%)	13 (100%)	99 (96%)	0.83
Spontaneous vaginal delivery [number (%)]	18 (67%)	5 (56%)	12 (92%)	71 (69%)	0.25
Cesarean section [number (%)]	9 (33%)	4 (44%)	1 (8%)	29 (28%)	0.24
Forceps delivery [number (%)]	0 (0%)	0 (0%)	0 (0%)	3 (3%)	0.69
Birth weight [grams, median (IQR)]	1410 (1170-1730)	1590 (1295-2451)	1980 (1840-2215)	2310 (1840-2570)	**<0.0001**
Apgar score <7; 5 minutes [number (%)]	1 (4%)	2 (22%)	1 (8%)	0 (0%)	**0.0005**
Apgar score <7; 10 minutes [number (%)]	0 (0%)	1 (11%)	1 (8%)	0 (0%)	**0.006**

CRP, C-reactive protein; IQR, interquartile range; PPROM, preterm prelabor rupture of membranes; WBC, white blood cells. Continuous variables, presented as median (interquartile range), were compared using a nonparametric Kruskal-Wallis *H* test. Categorical variables, presented as number (%), were compared using chi-square test. Statistically significant results are marked in bold.

**Fig 1:**
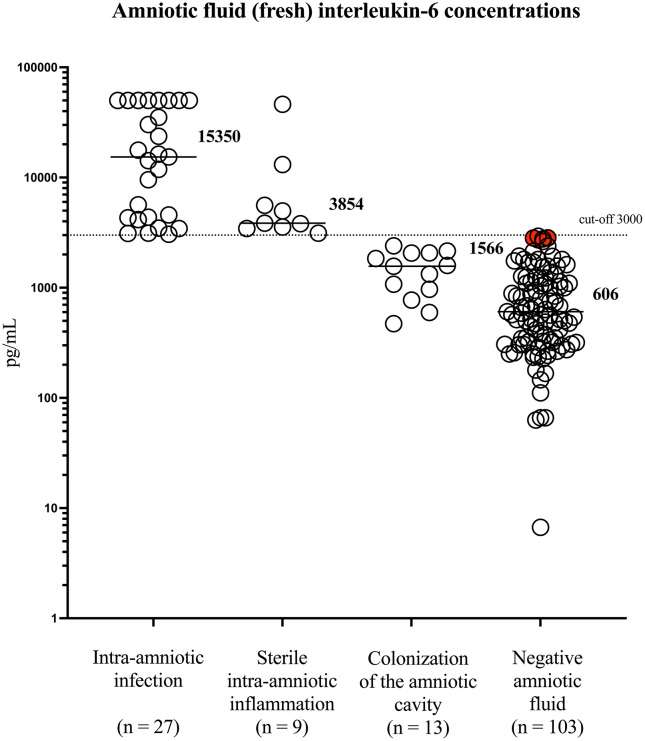
Comparison of interleukin-6 concentrations in fresh amniotic fluid among of women with preterm prelabor rupture of membranes with intra-amniotic infection, sterile intra-amniotic inflammation, colonization of the amniotic cavity, and negative amniotic fluid. Red circles indicated women who had intra-amniotic inflammation in the processed samples despite having negative amniotic fluid results in the fresh samples.

Concentrations of IL-6 in fresh amniotic fluid in women with intra-amniotic inflammation, sterile intra-amniotic inflammation, colonization of the amniotic cavity and negative amniotic fluid are shown in Fig 1.

IL-6 concentrations above the upper measuring limit (> 50,000 pg/mL) were observed in 8 of 152 fresh samples (5.3%) and 9 of 152 processed samples (5.9%).

The median storage duration of the processed amniotic fluid was 40 months (IQR 34–46). There was no difference in storage duration among groups (*p* = 0.22): intra-amniotic infection (median 39 months, IQR 34–45), sterile intra-amniotic inflammation (median 39 months, IQR 35–46**),** colonization of the amniotic cavity (median 45 months, IQR 37–51), and negative amniotic fluid (median 40 months, IQR 34–46).

### Comparison of IL-6 concentrations between fresh and processed amniotic fluid samples

**Fig 2:**
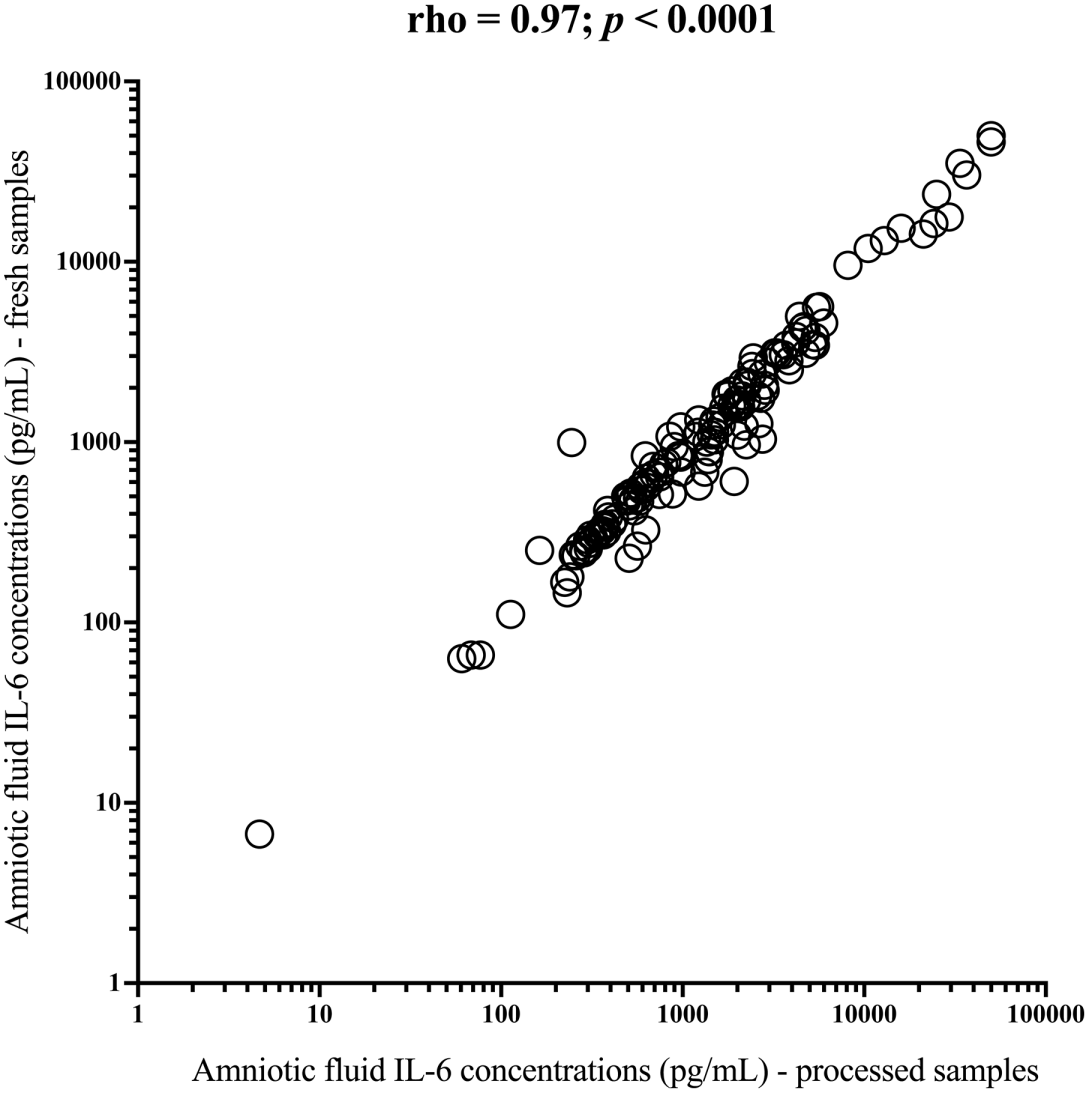
Correlation between amniotic fluid interleukin-6 concentrations measured in fresh and processed samples.

**Fig 3:**
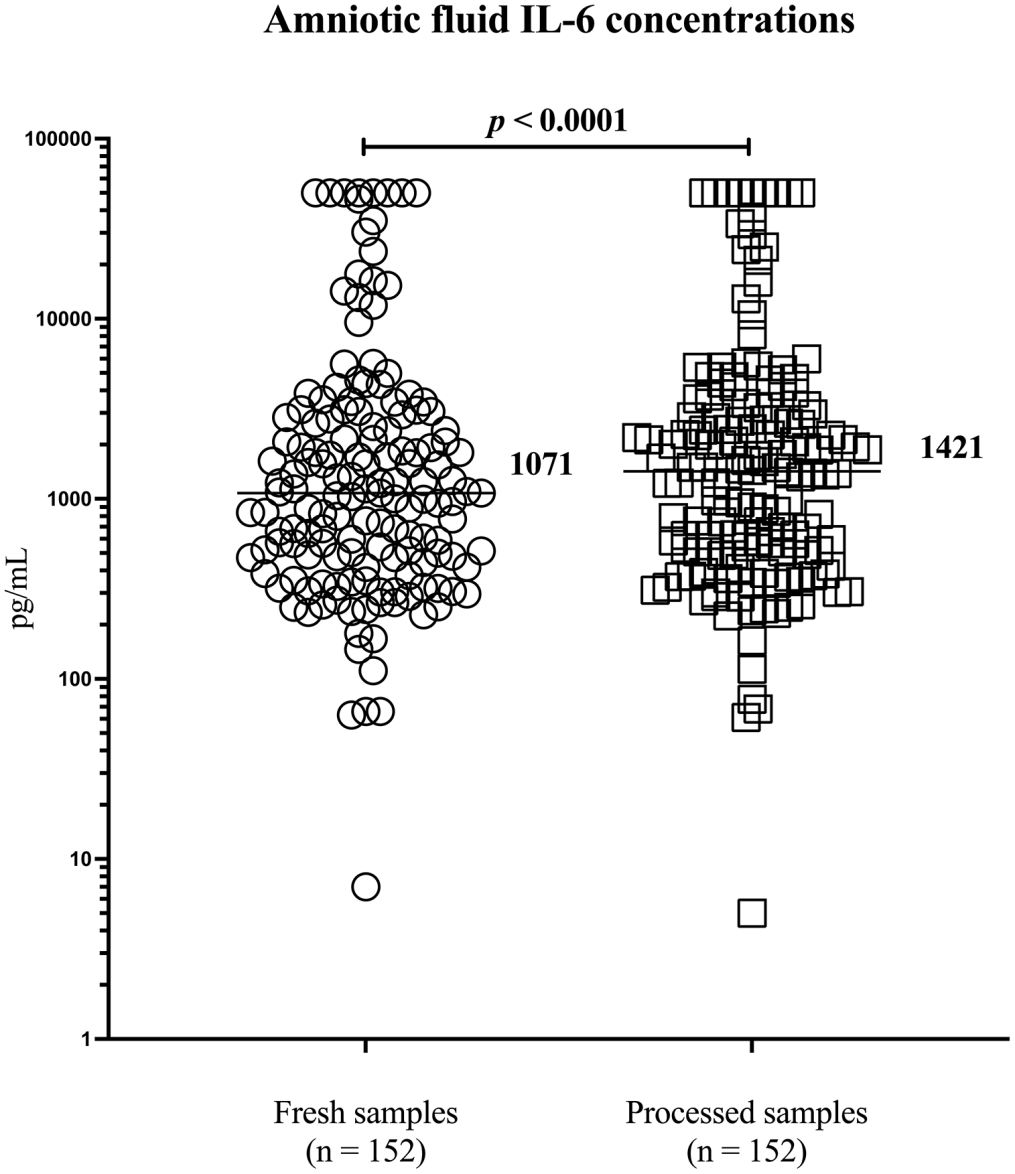
Comparison between amniotic fluid interleukin-6 concentrations in fresh and processed samples.

IL-6 concentrations measured in fresh and processed amniotic fluid samples were highly correlated (rho = 0.97; *p* < 0.0001; Fig 2). However, values were lower in fresh samples compared to processed samples (fresh: median 1,071 pg/mL, IQR 471−2,905 vs. processed: median 1,421 pg/mL, IQR 507−3,271; *p* < 0.0001; Fig 3).

**Fig 4:**
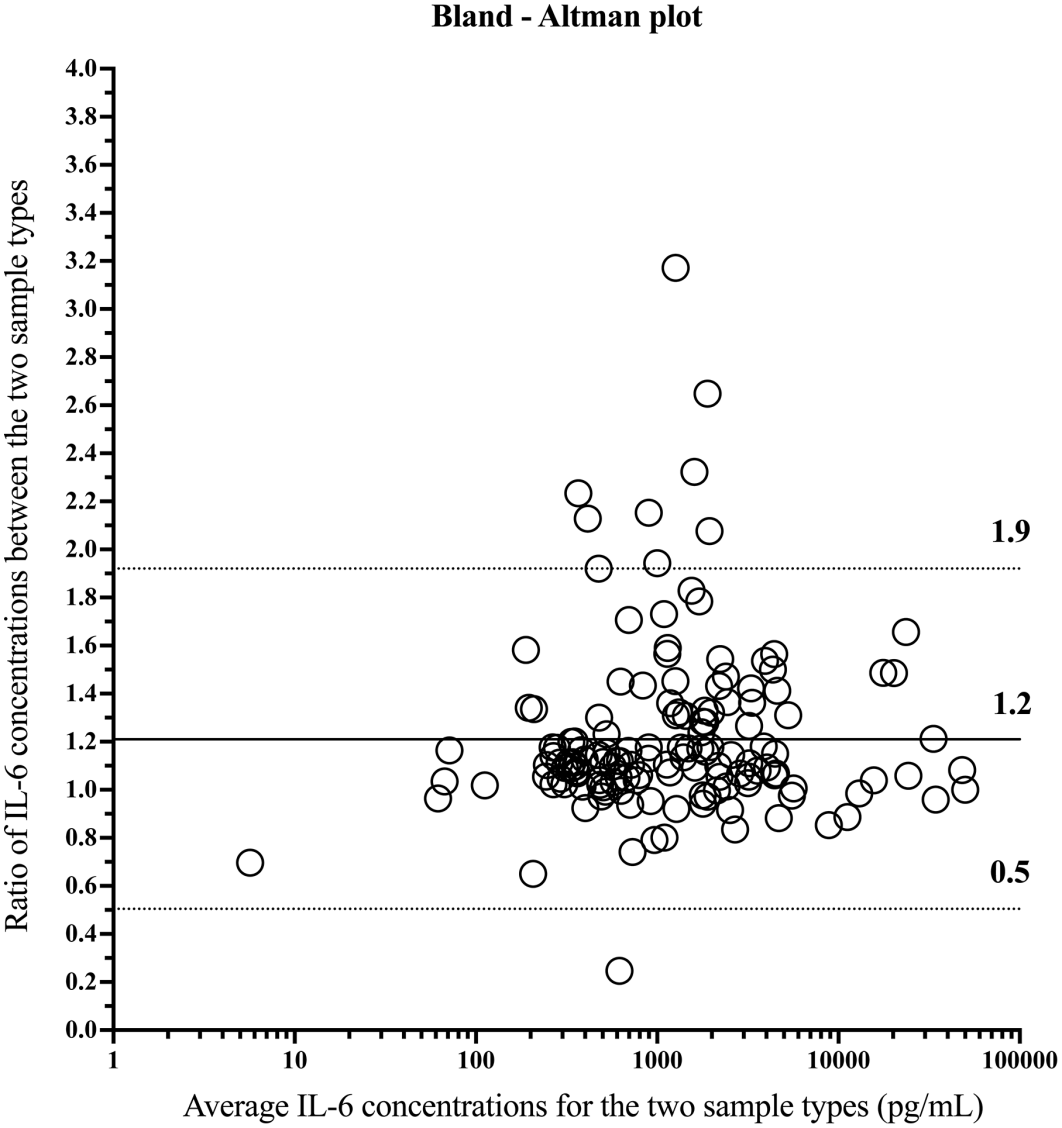
Bland-Altman plot comparing amniotic fluid interleukin-6 concentrations measured in fresh and processed samples.

Bland-Altman analysis using a ratio-based approach demonstrated a systematic bias toward higher IL-6 concentrations in processed samples, with a mean ratio of 1.2, indicating that processed samples yielded on average approximately 20% higher values than fresh samples (Fig 4). The limits of agreement ranged from 0.5 to 1.9, indicating that IL-6 concentrations in processed samples were generally between 50% lower and 90% higher than the corresponding concentrations in fresh samples.

When applying the validated diagnostic threshold of 3,000 pg/mL (established in processed samples) to fresh samples, three false-negative cases were observed (all with sterile intra-amniotic inflammation). In these cases, intra-amniotic inflammation was present, but IL-6 concentrations in fresh samples were below the threshold, despite corresponding processed values ≥ 3,000 pg/mL.

Sensitivity analyses excluding samples with IL-6 concentrations > 50,000 pg/mL did not alter the correlation (rho = 0.97; *p* < 0.0001), paired comparison (fresh: median 994 pg/mL, IQR 418−2,150 vs. processed: median 1,323 pg/mL, IQR 491−2,700; *p* < 0.0001), and Bland-Altman agreement between fresh and processed samples (mean ratio of 1.2, the limits of agreement from 0.5 to 1.9).

Using intra-amniotic inflammation in processed samples as the reference, the diagnostic performance of the 3,000 pg/mL threshold applied to fresh samples was as follows: sensitivity of 92% (36/39; 95% confidence interval [CI], 80–97), specificity 100% (103/103; 95% CI, 96–100), positive predictive value 100% (36/36; 95% CI, 90–100%), and negative predictive value 97% (103/106; 95% CI, 92–99).

## Discussion

In this study we demonstrate that IL-6 concentrations measured in amniotic fluid supernatant are consistently higher after a single freeze–thaw cycle than in matched fresh samples. Because IL-6 is a key biomarker of intra-amniotic inflammation, this finding may have direct implications for both clinical decision-making and research based on stored biological material.

Most published investigations on cytokine stability have focused on serum or plasma [22–25]. These studies generally suggest that IL-6 is relatively stable across several freeze–thaw cycles, although variability has been reported [22–25]. Some investigations observed little to no change in IL-6 concentrations even after multiple freeze–thaw events, whereas others documented gradual declines during long-term storage, particularly in amniotic fluid [22–27]. Overall, findings indicate that cytokine stability may depend on sample matrix, storage conditions, and analytical method, underscoring the need for validation in clinically relevant settings [22–25].

Our present results build on and extend previous work from our group investigating the impact of pre-analytical handling and storage on amniotic fluid IL-6 concentrations [14]. In an earlier study, we demonstrated that variations in sample processing, including latency time before centrifugation, centrifugation conditions, and addition of protease inhibitors, did not significantly influence IL-6 concentrations, although no direct comparison with fresh samples was performed [14]. Later, our group showed that IL-6 concentrations measured by a point-of-care IL-6 test (the lateral immunoflow assay) were lower in fresh amniotic fluid samples compared with their processed counterparts [28]. In that study, however, the fresh samples were analyzed without prior centrifugation [28]. This previous observation is in line with the result of the present study, where both types of samples underwent centrifugation.

Several biological and analytical factors may explain the observed increase in IL-6 concentrations in processed samples, which underwent a freeze-thaw cycle. First, extracellular vesicle disruption. Amniotic fluid is rich in exosomes and microvesicles capable of encapsulating or surface-binding cytokines [29,30]. Conventional pre-analytical centrifugation at low g-forces does not fully remove these vesicles [31]. Ice crystal formation and osmotic stress during freezing and thawing can rupture vesicles, releasing IL-6 into the soluble phase that is accessible to immunoassays [32,33]. Second, dissociation of protein complexes. IL-6 circulates partly bound to soluble IL-6 receptor or other carrier proteins [34]. Freezing can destabilize these complexes, expose epitopes and increase antibody recognition [35,36]. Last, matrix and assay effects. Physical changes of the matrix (pH, ionic strength, viscosity) and cryoconcentration may enhance antigen–antibody interactions or reduce inhibitory matrix effects [36,37]. Minor evaporation during freezing could further concentrate solutes if mixing is incomplete after thawing. These mechanisms are not mutually exclusive and may act synergistically.

In the present study, the diagnostic cut-off of 3,000 pg/mL for IL-6, originally validated in processed samples [14], did not fully translate to fresh specimens. Although correlation between fresh and processed values was excellent, a ratio-based Bland–Altman analysis demonstrated a systematic bias, with processed samples yielding approximately 20% higher concentrations. While the limits of agreement were relatively wide, the clinical impact of this variability is primarily confined to IL-6 concentrations near the diagnostic threshold. At concentrations far exceeding the threshold – clearly indicate intra-amniotic inflammation – proportional differences between fresh and processed samples are unlikely to affect clinical decision-making.

From a clinical perspective, our findings suggest that caution is warranted when applying diagnostic threshold for intra-amniotic inflammation derived exclusively from biobanked specimens. In our cohort, use of the 3,000 pg/mL threshold, validated in processed samples, resulted in three false-negative cases when applied to fresh samples, all corresponding to sterile intra-amniotic inflammation. Although the overall diagnostic performance of this threshold remained high, these missed cases underscore the risk of underdiagnosis when thresholds are not validated directly in fresh clinical material.

Given that individualized management of PPROM is based on the presence of intra-amniotic inflammation even a small number of missed cases may carry clinical significance. Such misclassification has the potential to influence clinical management, including antibiotic selection and the choice between expectant and active management, depending on gestational age and subsequent microbiological findings. These considerations highlight the clinical relevance of validating diagnostic cut-offs specifically for fresh amniotic fluid samples.

The strengths of this study are several. First, we used a paired design, directly comparing fresh and processed aliquots of the same amniotic fluid sample. Second, both fresh and processed samples were handled according to the standardized protocols and analyzed on the same automated electrochemiluminescence platform, thereby minimizing inter-individual and batch variability among the fresh and processed samples. Third, a relatively large cohort of women with one well-defined clinical phenotype of spontaneous preterm labor (PPROM) was included in the study. Finally, the study population was ethnically homogeneous (Caucasian), which reduced variability related to population-specific or genetic factors. Limitations should also be acknowledged. First, we evaluated only a single freeze–thaw cycle, while additional cycles or prolonged storage may produce different effects, including potential cytokine degradation. Second, pre-analytical handling of fresh and processed samples differed and may represent a potential source of bias. For examples, fresh samples were centrifuged promptly in the biochemistry laboratory but for a shorter duration than processed samples. Third, storage time of the processed samples was not uniform, ranging from 28 to 51 months, which could have affected cytokine stability. Fourth, we did not assess extracellular vesicles or IL-6–binding complexes, which may influence measured IL-6 concentrations and could help explain differences between fresh and processed specimens. Last, the exploratory nature of this study should be acknowledged. No a priori sample size or power calculation was performed for the agreement analyses, as no prior data were available to inform such calculations. Nevertheless, the relatively large number of paired samples strengthens the robustness of the observed associations.

In conclusion, IL-6 concentrations in fresh and processed amniotic fluid samples are highly correlated, but processed samples consistently yield higher values. This discrepancy is most critical for fresh samples with IL-6 concentrations just below the diagnostic threshold, which may be falsely classified as negative, underscoring the need to validate and possibly adjust cut-offs for fresh clinical specimens to ensure accurate diagnosis of intra-amniotic inflammation.
